# Dexmedetomidine versus Subanaesthetic Ketamine for Opioid-sparing Anaesthesia During Lumbar Spine Surgery Under a Standardised Surgical Pleth Index-guided Analgesic Protocol: A Randomised Controlled Trial

**DOI:** 10.4274/TJAR.2026.262419

**Published:** 2026-10-30

**Authors:** Gowthaman Arivuselvan, Srinivasan Swaminathan, Rajasekar Ramadurai, Prasanna Udupi Bidkar

**Affiliations:** 1 Jawaharlal Institute of Postgraduate Medical Education and Research (JIPMER) Department of Anaesthesiology and Critical Care, Puducherry, India

**Keywords:** SPI monitoring, adequacy of anaesthesia, dexmedetomidine, ketamine, lumbar spine surgery, opioid-sparing anaesthesia, QoR-15

## Abstract

**Objective:**

Lumbar spine surgery is associated with significant perioperative pain and increased opioid requirements. Objective nociception-guided opioid administration using the surgical pleth index (SPI) may facilitate individualised intraoperative analgesic titration. Dexmedetomidine and subanaesthetic ketamine are commonly used opioid-sparing adjuvants; however, their effects under SPI-guided analgesia remain inadequately studied.

**Methods:**

In this prospective randomised controlled trial, 50 adult patients undergoing lumbar spine surgery were allocated to 3 groups: dexmedetomidine (Group D), ketamine (Group K), and saline control (Group S). After prone positioning, Group D received a 0.5 µg kg^-1^ dexmedetomidine bolus followed by a 0.2 µg kg^-1^ h infusion; Group K received a 0.5 mg kg^-1^ ketamine bolus followed by a 0.2 mg kg^-1^ h^-1^ infusion; and Group S received saline. Intraoperative fentanyl (0.5 µg kg^-1^) was administered when SPI exceeded 50, as indicated by Adequacy of Anaesthesia monitoring, allowing objective nociception-guided opioid titration. The primary outcome was intraoperative supplemental fentanyl consumption. Secondary outcomes included extubation time, postoperative pain scores, 24-hour fentanyl consumption, and Quality of Recovery (QoR-15) scores.

**Results:**

Cumulative intraoperative supplemental fentanyl consumption differed among the groups (*P *< 0.001), with lower requirements in Groups D (150±45.9 µg) and K (161.7±35.4 µg) than in Group S (220.5±42.6 µg). In contrast, no difference was observed between Groups D and K. Twenty-four–hour postoperative fentanyl consumption also differed significantly among the groups (*P* < 0.001), with lower consumption in Group D than in Group S, and intermediate consumption in Group K. Extubation time was comparable among the groups. Dexmedetomidine resulted in lower postoperative visual analogue scale scores than saline at all time points; scores also differed between dexmedetomidine and ketamine at 1 hour and between ketamine and saline at 12 hours. QoR-15 scores differed significantly among the groups, with both active treatment groups showing improved recovery compared with the saline group.

**Conclusion:**

Within a standardised SPI-guided analgesic protocol, both dexmedetomidine and subanaesthetic ketamine reduced intraoperative opioid requirements without prolonging extubation time compared with saline during lumbar spine surgery. Dexmedetomidine was associated with lower postoperative opioid consumption and improved postoperative recovery compared with saline. Both interventions demonstrated comparable intraoperative opioid-sparing efficacy.

**Main Points**

• A standardised surgical pleth index (SPI)-guided analgesic protocol was used to facilitate predefined intraoperative opioid titration and to compare the opioid-sparing effects of dexmedetomidine and ketamine with those of saline.

• Within a standardised SPI-guided analgesic protocol, both dexmedetomidine and ketamine significantly reduced intraoperative opioid requirements compared with saline.

• Dexmedetomidine was associated with lower postoperative opioid consumption and favourable recovery outcomes compared with saline.

• Postoperative pain scores differed significantly among the groups at all assessed time points, with dexmedetomidine demonstrating consistently lower pain scores than saline.

## Introduction

Lumbar spine surgeries are frequently associated with significant postoperative pain due to extensive tissue dissection and muscle retraction required for adequate surgical exposure.^1, 2^ In addition, many patients present with chronic pain and preoperative opioid use, predisposing them to opioid tolerance and increased perioperative opioid requirements. This escalation in opioid consumption may lead to an increased incidence of opioid-related adverse effects, such as postoperative nausea and vomiting, respiratory depression, sedation, and delayed recovery.^3^ To mitigate these challenges, opioid-sparing strategies have been increasingly incorporated into multimodal anaesthesia. Dexmedetomidine and subanaesthetic ketamine are widely used anaesthetic adjuvants with established analgesic properties and the potential to reduce perioperative opioid requirements.^4, 5, 6, 7^ However, a significant methodological challenge remains: most previous studies evaluating these agents have relied on indirect, subjective parameters-such as changes in heart rate and blood pressure-to guide intraoperative analgesic administration. These parameters may be influenced by several perioperative factors, including anaesthetic depth, intravascular volume status, and surgical stimulation, thereby limiting their reliability as indicators of nociception.^2^ The surgical pleth index (SPI) (GE Healthcare, Helsinki, Finland) is a significant methodological advancement, providing an objective tool for monitoring nociception based on photoplethysmographic waveform analysis and reflects the balance between nociception and antinociception during general anaesthesia.^8, 9, 10^ By utilising SPI, clinicians can move toward a physiologically tailored approach that enables real-time assessment of nociceptive responses and facilitates precise and individualised titration of analgesics, particularly opioids. While the individual efficacy of dexmedetomidine and ketamine is documented, their performance within an objective, SPI-guided titration framework requires further exploration. This study was designed to compare the opioid-sparing effects of dexmedetomidine and subanaesthetic ketamine using a standardised SPI-guided analgesic protocol during lumbar spine surgery. The primary objective was to compare the cumulative intraoperative supplemental fentanyl consumption between the groups. The secondary objectives included comparisons of extubation time, postoperative pain scores, total fentanyl consumption during the first 24 h, and the Quality of Recovery (QoR-15), assessed using the QoR-15 score. We hypothesised that dexmedetomidine and ketamine would reduce intraoperative opioid requirements during SPI-guided analgesic administration, with potential differences in postoperative recovery outcomes between the two agents.

## Methods

This prospective randomised controlled trial in adult patients undergoing lumbar spine surgery was conducted after obtaining approval from the Jawaharlal Institute of Postgraduate Medical Education and Research Institute Ethics Committee (approval no: JIP/IEC/2022/09, date: 26.03.2022) and registration with the Clinical Trial Registry of India (CTRI/2023/01/048710). Written informed consent was obtained from all the participants. Fifty patients, aged 18-70 years and of either sex, with American Society of Anesthesiologists physical status I-II were included. Patients with known allergies to study drugs, increased intracranial or intraocular pressure, ischemic heart disease, uncontrolled hypertension, psychiatric illness, seizure disorder, or significant hepatic or renal dysfunction were excluded.

Participants were randomly allocated to 3 groups (Groups D, K, and S) using a computer-generated block randomisation sequence with block sizes of 3 and 6. Randomisation and allocation concealment were achieved using sequentially numbered, opaque, sealed envelopes. Study drugs were prepared by an independent investigator who was not involved in intraoperative management, postoperative assessment, or data analysis. Patients and postoperative outcome assessors remained blinded to treatment allocation. Owing to the nature of the study drug administration, the attending intraoperative anaesthesiologist was aware of group allocation and, therefore, was not blinded. To minimise performance bias, intraoperative fentanyl administration was standardised using a predefined SPI-guided analgesic protocol. Data analysis was performed using coded group allocation.

Upon arrival in the operating room, standard monitoring, including electrocardiography, pulse oximetry, and non-invasive blood pressure monitoring, was instituted, and intravenous access was secured. Entropy sensors were applied prior to induction. Glycopyrrolate (0.2 mg) was administered intravenously, followed by induction of anaesthesia with fentanyl (2 µg kg^-1^) and propofol (2 mg kg^-1^). Tracheal intubation was facilitated with vecuronium (0.1 mg kg^-1^). Anaesthesia was maintained with isoflurane in an oxygen-air mixture. Supplemental vecuronium was administered in 1-mg incremental doses at approximately 40-minute intervals, or earlier if clinical signs of recovery from neuromuscular blockade, such as spontaneous respiratory efforts or patient movement, were observed. The same neuromuscular blockade protocol was followed for all patients throughout the study. Intravenous paracetamol (1 g) was administered prior to the skin incision.

After positioning the patient prone, the study drug was administered as a 10-min loading dose, followed by continuous infusion. Group D received dexmedetomidine (0.5 µg kg^-1^) as a bolus, followed by a 0.2 µg kg^-1^ h^-1^ infusion. Group K received ketamine 0.5 mg kg^-1^ as a bolus, followed by a 0.2 mg kg^-1^ h^-1^ infusion. Group S received an equivalent volume of 0.9% saline solution. Infusion was continued until muscle closure. Dosing regimens of dexmedetomidine and ketamine were selected based on published studies demonstrating their efficacy as opioid-sparing anaesthetic adjuvants. Given the differing pharmacological mechanisms of these agents, direct dose equivalence was not feasible; therefore, clinically accepted subanaesthetic doses were utilised. The study was designed to evaluate opioid consumption under a standardised SPI-guided analgesic protocol rather than to establish definitive pharmacologic superiority between dexmedetomidine and ketamine.

Adequacy of anaesthesia (AoA) monitoring was performed using a GE Healthcare CARESCAPE B650 monitor (GE Healthcare, Helsinki, Finland), equipped with the E-Entropy module for entropy monitoring and the E-PreSTN module for SPI monitoring. State entropy was maintained between 40 and 60 throughout the surgery by adjusting the inhalational anaesthetic concentration to ensure a standardised anaesthetic depth. The integration of entropy and SPI constitutes AoA monitoring (Figure 1A).

SPI values were continuously monitored intraoperatively as part of the standardised analgesic protocol. Baseline SPI was recorded after induction and before administration of the loading dose of the study drug, while the patient was under stable anaesthesia and prior to surgical incision; stable anaesthesia was defined as the absence of surgical stimulation and stable hemodynamics for at least 2 min. An SPI range of 20-50 was considered acceptable (Figure 1B). If SPI exceeded 50 for more than 1 min, fentanyl at 0.5 µg kg^-1^ was administered intravenously. SPI was reassessed 5 min after each fentanyl bolus, and an additional fentanyl bolus of 0.5 µg kg^-1^ was administered only if SPI remained above 50. Potential confounding factors affecting SPI interpretation, including electrocautery, patient movement, arrhythmias, vasoactive drug administration, abrupt changes in anaesthetic depth, and haemodynamic instability, were identified before clinical intervention. During such events, SPI values were not used to guide fentanyl administration. Opioid titration was deferred until the confounding factor had resolved and a stable SPI signal had been re-established under steady-state anaesthetic and ventilatory conditions. Thus, while dexmedetomidine and ketamine were administered as fixed-dose infusions, opioids were titrated objectively using SPI-based monitoring. The attending anaesthesiologist recorded the SPI values and fentanyl administration as part of routine intraoperative monitoring. Intraoperative fentanyl consumption was defined as the cumulative supplemental fentanyl administered in response to the SPI-guided protocol after induction; the initial fentanyl dose of 2 µg kg^-1^ administered for induction was excluded from this calculation.

The study drug infusion was discontinued at the end of surgery. Residual neuromuscular blockade was reversed with intravenous neostigmine and glycopyrrolate, in accordance with institutional practice. As objective neuromuscular monitoring was not used, extubation was performed after the fulfilment of standard clinical criteria, including adequate spontaneous ventilation, purposeful motor response, satisfactory airway-protective reflexes, and the ability to obey verbal commands. Extubation time was defined as the interval from the administration of reversal agents to successful extubation. Postoperative analgesia was provided via intravenous patient-controlled delivery of fentanyl (bolus dose 30 µg, lockout interval 10 min, no background infusion, maximum 150 µg/h). All patients received intravenous paracetamol (1 g every 8 h). Postoperative pain intensity was assessed using a visual analogue scale (VAS) at 1, 6, 12, and 24 hours after surgery. The QoR-15 score was assessed at 24 hours using the QoR-15 questionnaire.

The sample size was calculated using OpenEpi software (Version 3.01, Atlanta, GA, USA) based on a previous study by Mitra et al.^2^, which reported a mean difference in fentanyl consumption of 14.3 µg (standard deviation 12 µg). A sample size of 15 patients per group was required to achieve a power of 80% at a significance level of 0.05. A total of 50 patients were included to account for potential attrition. Data are presented as the mean ± standard deviation. The sample size was calculated to detect overall differences in opioid consumption between study groups under SPI-guided analgesic administration. It was not specifically powered for a definitive head-to-head comparison between dexmedetomidine and ketamine.

### Statistical Analysis

Statistical analyses were performed using IBM SPSS Statistics version 19 (IBM Corp., Armonk, NY, USA). Primary analyses focused on differences in opioid consumption under the standardised SPI-guided analgesic protocol. The normality of the data was assessed using the Shapiro-Wilk test, which confirmed that continuous variables were normally distributed. Continuous variables were expressed as mean ± standard deviation and compared using one-way analysis of variance (ANOVA). When the overall ANOVA was statistically significant, Bonferroni-corrected post-hoc pairwise comparisons were performed to identify between-group differences while controlling the Type I error rate. Categorical variables were expressed as frequencies and percentages and analysed using the chi-square test or Fisher’s exact test, as appropriate. A *P* value <0.05 was considered statistically significant.

## Results

Fifty-five patients were assessed for eligibility; 5 were excluded before randomisation because 3 did not meet the inclusion criteria and 2 declined to participate. The remaining 50 patients were randomised into 3 groups, all of whom were included in the final analysis (Figure 2). Baseline demographic characteristics, including age, sex, body mass index, weight, and American Society of Anesthesiologists physical status, were comparable among the groups. Surgical characteristics, including operative duration and distribution of procedure types, were also comparable among the groups (Table 1).

Under the standardised SPI-guided analgesic protocol, cumulative intraoperative supplemental fentanyl consumption differed significantly among the 3 groups (one-way ANOVA, *P* < 0.001). Mean intraoperative supplemental fentanyl consumption was 150±45.9 µg in Group D, 161.7±35.4 µg in Group K, and 220.5±42.6 µg in Group S. Bonferroni-corrected post-hoc analysis demonstrated that intraoperative fentanyl consumption was significantly lower in Group D than in Group S (*P* < 0.001), and in Group K than in Group S (*P* < 0.001). No significant difference was observed between Groups D and K (*P*=1.000) (Table 2).

Total 24-hour postoperative fentanyl consumption also differed significantly among the groups (one-way ANOVA, *P* < 0.001). Mean postoperative fentanyl consumption was 271.3±118.5 µg in Group D, 374.1±80.8 µg in Group K, and 444.1±119.7 µg in Group S. Bonferroni-corrected post-hoc analysis demonstrated significantly lower postoperative fentanyl consumption in Group D than in Group S (*P* < 0.001) and than in Group K (*P*=0.026). In contrast, the difference between Group K and Group S was not statistically significant (*P*=0.192) (Table 2).

Extubation times were comparable among the groups (*P*=0.08) (Table 2). Postoperative VAS scores differed significantly among the groups at all assessed time points. Bonferroni-corrected post-hoc analysis showed that Group D had lower VAS scores than Group S throughout the 24-h postoperative period. Group D also demonstrated lower VAS scores than Group K at 1 h, whereas Group K demonstrated lower VAS scores than Group S at 12 h. No other pairwise differences were statistically significant (Figure 3 and Table 3).

QoR-15, assessed using the QoR-15 questionnaire, differed significantly among the groups (one-way ANOVA, *P* < 0.001). Mean QoR-15 scores were 123±6.9 in Group D, 117±6.8 in Group K, and 109±10.0 in Group S. Bonferroni-corrected post-hoc analyses demonstrated that QoR-15 scores were significantly higher in Group D than in Group S (*P* < 0.001) and in Group K than in Group S (*P*=0.017). In contrast, no significant difference was observed between Groups D and K (*P*=0.117) (Table 2).

## Discussion

The present study demonstrated that, within a standardised SPI-guided opioid titration protocol, both dexmedetomidine and subanaesthetic ketamine significantly reduced intraoperative supplemental fentanyl requirements compared with saline during elective lumbar spine surgery. Although both active interventions exhibited comparable intraoperative opioid-sparing efficacy, dexmedetomidine was associated with lower postoperative fentanyl consumption and improved QoR-15 compared with saline. The objective of this study was to compare the opioid-sparing effects of dexmedetomidine and ketamine using a standardised SPI-guided analgesic protocol rather than evaluate the effectiveness of SPI monitoring itself.

Both dexmedetomidine and ketamine reduce opioid requirements through distinct pharmacological mechanisms. Dexmedetomidine, a highly selective α_2_-adrenergic receptor agonist, produces analgesia by inhibiting norepinephrine release within the locus coeruleus and dorsal horn of the spinal cord, thereby attenuating sympathetic activation and nociceptive transmission.^11^ In contrast, ketamine primarily exerts its analgesic effects through N-methyl-D-aspartate (NMDA) receptor antagonism, reducing central sensitisation and limiting opioid-induced hyperalgesia.^12^ Despite these differing mechanisms, both agents produced comparable reductions in intraoperative supplemental fentanyl consumption when opioid administration was standardised using a predefined SPI threshold, suggesting that either adjunct can effectively reduce intraoperative opioid requirements during lumbar spine surgery.

An important consideration when interpreting these findings is that SPI is derived from the normalised heartbeat interval and pulse plethysmographic amplitude, both of which may be influenced by autonomic nervous system activity and nociceptive stimulation.^13^ As dexmedetomidine and ketamine have differing effects on sympathetic tone, a potential influence on SPI values cannot be entirely excluded. However, the same predefined SPI threshold and opioid titration protocol were applied across all study groups, and potential confounding factors were addressed before fentanyl administration. Therefore, SPI-guided opioid titration provided a standardised approach to comparing the study interventions, although the drugs’ autonomic effects on SPI should be considered when interpreting the findings.

Our findings are consistent with those of Andleeb et al.,^14^ who reported comparable intraoperative opioid-sparing effects of dexmedetomidine and subanaesthetic ketamine during elective spine surgery using a propofol-fentanyl total intravenous anaesthesia (TIVA) technique. In contrast, Khalil et al.^15^ observed significantly lower intraoperative opioid requirements with dexmedetomidine than with ketamine during bariatric surgery. A plausible explanation for this discrepancy lies in differences in anaesthetic technique. Khalil et al.^15^ employed propofol-based TIVA, whereas anaesthesia in the present study was maintained with isoflurane. Volatile anaesthetic agents influence peripheral vascular tone and photoplethysmographic waveform characteristics differently from propofol, potentially altering SPI behaviour and subsequent opioid titration.^16^Differences in patient populations, surgical procedures, and infusion regimens may have further contributed to the variation in findings between the 2 studies. Furthermore, unlike bariatric surgery, lumbar spine procedures involve prolonged muscle dissection, tissue retraction, and instrumentation, resulting in sustained nociceptive stimulation throughout surgery. Under these circumstances, standardised SPI-guided opioid titration may reduce variability in opioid administration by providing a predefined physiological target for analgesic supplementation, thereby allowing the opioid-sparing effects of dexmedetomidine and ketamine to be evaluated under similar intraoperative conditions.

Postoperative pain scores progressively decreased in all groups over the 24-h assessment period. Dexmedetomidine was associated with lower VAS scores than saline throughout the postoperative period and showed an additional early difference compared with ketamine at 1 h. This profile may reflect the sustained analgesic and sympatholytic effects of dexmedetomidine that extend into the postoperative period. Ketamine, despite its NMDA-receptor antagonism and antihyperalgesic effects, demonstrated a less consistent postoperative pain profile at the infusion dose used in this study. Mitra et al.^2^ similarly reported favourable postoperative analgesic effects of dexmedetomidine compared with ketamine in spine surgery. Nikoubakht et al.^4^ reported reduced postoperative pain and opioid requirements with both dexmedetomidine and ketamine compared with control patients undergoing lumbar spine surgery. Differences in postoperative analgesic techniques, including continuous fentanyl infusion with rescue pethidine in their study versus patient-controlled fentanyl analgesia in ours, may account for variations in the observed postoperative pain profiles.

Although intraoperative opioid-sparing efficacy was comparable between dexmedetomidine and ketamine, dexmedetomidine was associated with lower 24-hour postoperative fentanyl consumption. This finding is biologically plausible, as dexmedetomidine provides sustained modulation of both spinal and supraspinal nociceptive pathways while attenuating sympathetic activation throughout the perioperative period.^17^ Ketamine primarily reduces central sensitisation and opioid-induced hyperalgesia, but its postoperative analgesic benefit may diminish after discontinuation of the infusion at muscle closure.^18^These pharmacological differences may explain the superior postoperative opioid-sparing effect observed with dexmedetomidine despite similar cumulative intraoperative supplemental fentanyl requirements.

Extubation times were comparable among the 3 groups despite dexmedetomidine’s sedative properties. This observation is consistent with previous reports demonstrating that low-dose dexmedetomidine infusions do not necessarily delay emergence when appropriate neuromuscular reversal and standardised extubation criteria are employed. In the present study, all patients received the same neuromuscular blockade protocol and reversal regimen, and extubation was performed according to predefined clinical criteria, thereby minimising systematic differences between the study groups. Nevertheless, objective neuromuscular monitoring was not employed, and this should be considered when interpreting extubation-related outcomes.

QoR, assessed using the QoR-15 questionnaire, was significantly better in both active intervention groups compared with the saline group, and the highest scores were observed in the dexmedetomidine group. Beyond reducing opioid consumption, dexmedetomidine may improve postoperative recovery through its favourable effects on analgesia, sleep quality, anxiolysis, and opioid-related adverse effects, without causing clinically significant respiratory depression. These findings are consistent with previous studies by Kim et al.^19^ and Lee et al.^20^ Similarly, the improvement observed with ketamine is in agreement with the findings of Pereira et al.^21^ Importantly, the improvement in QoR-15 scores with dexmedetomidine compared with saline exceeded the reported minimal clinically important difference (MCID) of 6-8 points, indicating that the observed benefit was not only statistically significant but also clinically meaningful.^22, 23^ Although the mean QoR-15 score was 6 points higher with dexmedetomidine than with ketamine, meeting the updated 6-point MCID, this between-group difference should be interpreted cautiously, as the MCID was established primarily to reflect clinically important change at the individual-patient level.^23^

Beyond the immediate perioperative outcomes observed in this study, these findings have potential implications for perioperative analgesic strategies and their broader clinical application. The present findings also have potential implications for enhanced recovery pathways. The use of opioid-sparing adjuvants such as dexmedetomidine or subanaesthetic ketamine within a standardised SPI-guided analgesic protocol may facilitate individualised intraoperative opioid administration while reducing perioperative opioid exposure. Dexmedetomidine was associated with lower postoperative opioid consumption and improved QoR-15, suggesting that it may be a useful adjunct in settings where enhanced postoperative recovery is a priority.

Lumbar spine surgery encompasses procedures that vary in the number of spinal levels operated on, the extent of instrumentation, the degree of muscle dissection, and the duration of tissue retraction, all of which influence the intensity and duration of intraoperative nociceptive stimulation. More extensive procedures may generate greater afferent nociceptive input, resulting in higher analgesic requirements during surgery.^24^ Although the overall distributions of procedure types and operative durations were comparable among the study groups, these procedure-specific factors were not quantified and may have contributed to variability in perioperative opioid requirements. Nevertheless, the standardised SPI-guided opioid titration protocol ensured that fentanyl administration followed a predefined criterion uniformly across all study groups, thereby reducing, although not eliminating, the influence of surgical heterogeneity on intraoperative analgesic management.

Although this study was limited to lumbar-spine surgery, the observed opioid-sparing effects of dexmedetomidine and subanaesthetic ketamine may be broadly relevant to other surgical procedures that involve substantial nociceptive stimulation. Previous studies have similarly demonstrated improved intraoperative opioid titration with SPI-guided protocols across diverse surgical populations.^13, 25^ However, the present study was designed to compare adjunctive analgesic strategies under a standardised SPI-guided protocol, rather than to establish the superiority of SPI-guided analgesia over conventional analgesic management. Further multicentre studies across different surgical populations and anaesthetic techniques are warranted to establish the broader generalizability of these findings.

### Study Limitations

This study has certain limitations. First, the sample size was not specifically powered for a definitive head-to-head comparison between dexmedetomidine and ketamine. Second, the attending anaesthesiologist was not blinded to group allocation and was responsible for recording SPI values and administering intraoperative fentanyl. Although predefined SPI thresholds and a fixed fentanyl dosing protocol were used, this may have introduced performance or measurement bias. Third, objective neuromuscular monitoring was not employed, and cumulative supplemental vecuronium doses were not recorded, both of which may have influenced extubation time. Although procedure types and operative durations were comparable, surgical complexity, including the number of levels operated on, the extent of instrumentation, and tissue handling, was not specifically quantified and may have contributed to variability in nociceptive stimulation and perioperative opioid requirements.

In addition, dexmedetomidine and ketamine have distinct pharmacological mechanisms and opposing effects on autonomic tone, with no established dose equivalence between them. Although the same predefined SPI-guided opioid titration protocol was applied uniformly across all groups, these pharmacological differences should be considered when interpreting comparisons between the active interventions.

## Conclusion

Within a standardised SPI-guided analgesic protocol, both dexmedetomidine and subanaesthetic ketamine reduced cumulative intraoperative supplemental opioid requirements without prolonging extubation time compared with saline during lumbar spine surgery. Dexmedetomidine was associated with lower postoperative fentanyl consumption and improved postoperative recovery compared with saline, whereas both active interventions demonstrated comparable intraoperative opioid-sparing efficacy. As SPI-guided opioid titration served as a standardised analgesic protocol throughout the study, these findings reflect the comparative effects of the study interventions rather than the independent clinical effectiveness of SPI-guided analgesia. Further adequately powered studies are warranted to evaluate the role of SPI-guided opioid titration and to compare opioid-sparing adjuncts across different surgical populations.

## Ethics

**Ethics Committee Approval: **This prospective randomised controlled trial in adult patients undergoing lumbar spine surgery was conducted after obtaining approval from the Jawaharlal Institute of Postgraduate Medical Education and Research Institute Ethics Committee (approval no: JIP/IEC/2022/09, date: 26.03.2022) and registration with the Clinical Trial Registry of India (CTRI/2023/01/048710).

**Informed Consent:** Written informed consent was obtained from all the participants.

## Figures and Tables

**Figure 1:**
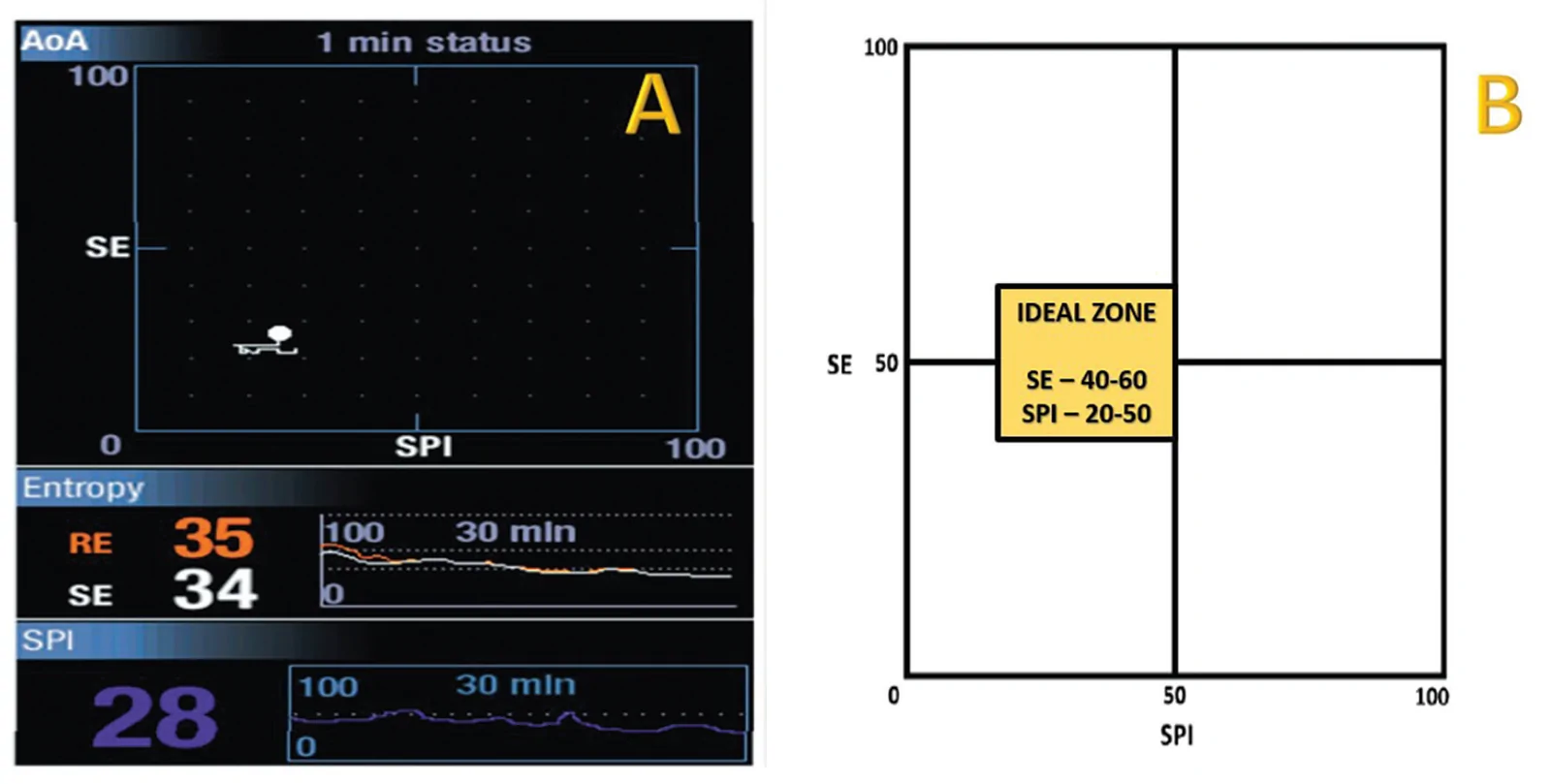
A) Adequacy of Anaesthesia (AoA™) monitoring display showing the integration of state entropy (SE) and surgical pleth index (SPI) during general anaesthesia; B) Schematic representation of the ideal zone in AoA display.

**Figure 2:**
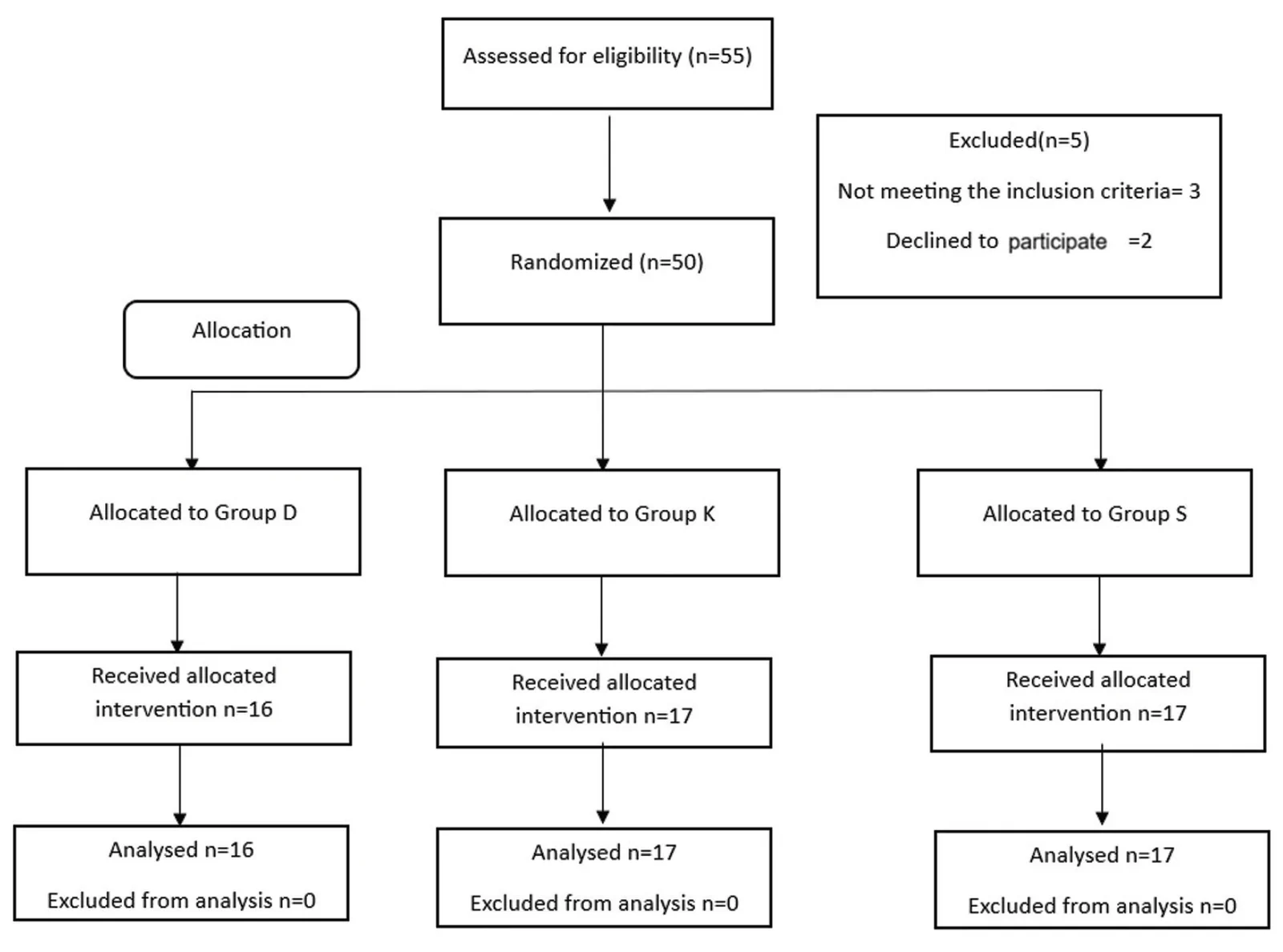
CONSORT diagram.

**Figure 3:**
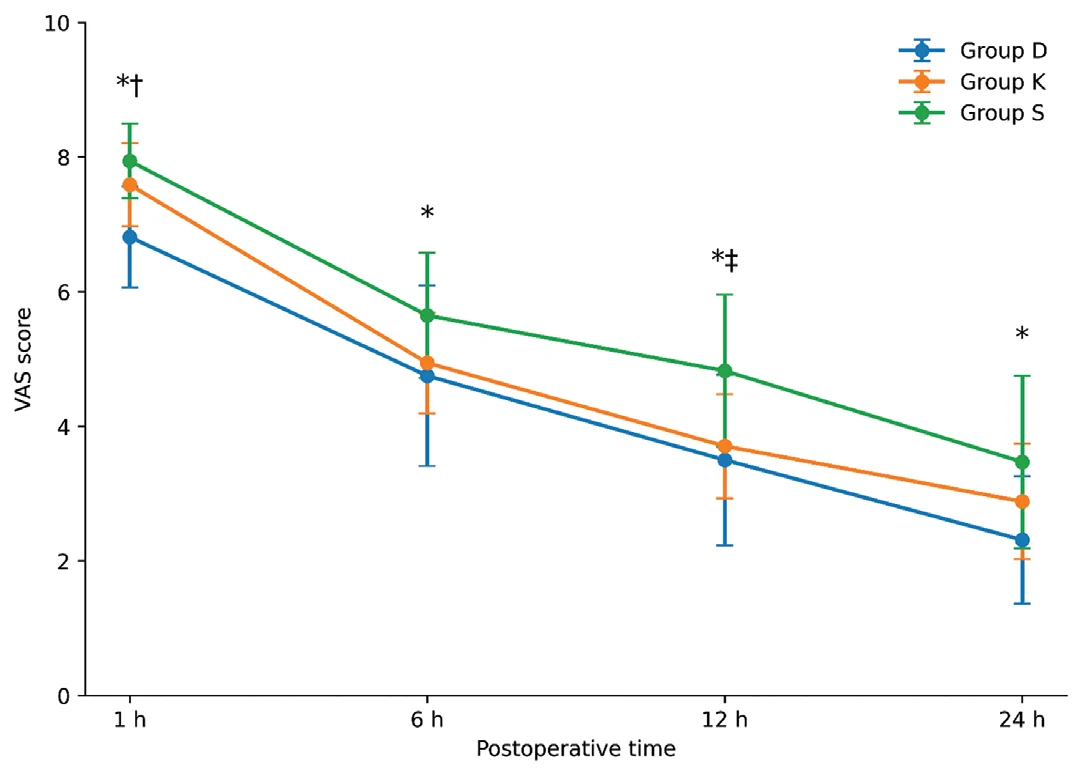
Postoperative visual analogue scale (VAS) scores among the 3 study groups over 24 hours. Data are presented as mean ± standard deviation. *P* < 0.05 for Group D versus Group S; †*P* <0.05 for Group D versus Group K; ‡*P* < 0.05 for Group K versus Group S, based on Bonferroni-corrected post-hoc comparisons. Group D, dexmedetomidine; Group K, ketamine; Group S, saline.

**Table 1. Demographic Parameters of the Study Population:** 

**Baseline characteristics**		**Group D (n = 16)**	**Group K (n = 17)**	**Group S (n = 17)**	***P* value**
**Age** **(years)**		45.6±15.78 (37.15-53.97)	41±12.6 (34.52-47.48)	45.6±14.3 (38.26-53.04)	0.56
**Gender** **n** **(%)**	Male	12 (75.0%)	8 (47.1%)	10 (58.8%)	0.28
	Female	4 (25.0%)	9 (52.9%)	7 (41.2%)	
**Weight** **(kg)**		64.9±8.41 (60.46-69.42)	62.9±5.2 (60.24-65.64)	66.3±5.62 (63.46-69.24)	0.32
**BMI** **(kg/m^2^)**		24.6±3.46 (22.85-26.54)	24±1.41 (23.36-24.81)	25.05±2.35 (23.82-26.29)	0.54
**ASA** **n** **(%)**	Class 1	11 (68.8%)	13 (76.5%)	13 (76.5%)	0.99
	Class 2	5 (31.2%)	4 (23.5%)	4 (23.5%)	
**Operative** **duration** **(min)**		282.6±65.5 (247.7-317.4)	280.9±63.7 (248.2-313.7)	277.5±66.1 (243.5-311.5)	0.97
**Procedure** **type** **n** **(%)**					
Instrumentation (fusion/fixation)		11 (68.8%)	11 (64.7%)	10 (58.8%)	0.97
Discectomy		2 (12.5%)	2 (11.8%)	3 (17.6%)	
Decompression		3 (18.8%)	4 (23.5%)	4 (23.5%)	

Values expressed as mean ± standard deviation (95% CI) and frequency (n). Continuous variables were analysed using one-way ANOVA, and categorical variables were analysed using chi-square or Fisher’s exact test

A *P* value <0.05 was considered statistically significant

D, dexmedetomidine; K, ketamine; S, saline; ANOVA, analysis of variance; ASA, American Society of Anesthesiologists; BMI, body mass index; CI, confidence interval

**Table 2. Comparison of Perioperative Fentanyl Requirement, Extubation Time, and QoR-15 Scores Between the 3 Groups:** 

**Variable**	**Group D(n = 16)**	**Group K(n = 17)**	**Group S(n = 17)**	**ANOVA*P* value**	**Bonferroni-corrected post-hoc *P* values**		
					**D vs. K**	**D vs. S**	**K vs. S**
**Intraoperative** **supplemental** **fentanyl** **requirement** **(µg)**	150±45.9 (125.55 - 174.45)	161.7±35.4 (143.56-179.96)	220.5±42.6 (198.66 - 242.51)	**<0.001**	1	**<0.001**	**<0.001**
**Postoperative** **fentanyl** **requirement** **(µg)**	271.3±118.5 (208.1-334.4)	374.1±80.8 (332.58-415.65)	444.1±119.7 (382.55-505.69)	**<0.001**	**0.026**	**<0.001**	0.192
**Extubation** **time** **(Mins)**	10.9±4.1 (8.72-13.03)	8.8±3.1 (7.21-10.44)	8.6±1.9 (7.67-9.63)	0.08	-	-	-
**QoR-15** **score**	123.6±6.9 (119.93-127.32)	117.6±6.8 (114.16-121.13)	109.6±10.1 (104.42-114.76)	**<0.001**	0.117	**<0.001**	**0.017**

Values expressed as mean ± standard deviation (95% CI). Continuous variables were analysed using one-way ANOVA followed by a Bonferroni post-hoc test. A *P* value <0.05 was considered statistically significant

D, dexmedetomidine; K, ketamine; S, saline; ANOVA, analysis of variance; Qor-15, quality of recovery

**Table 3. Comparison of Postoperative Visual Analogue Scale Scores Among the 3 Groups Over 24 Hours:** 

**Time point**	**Group D(n = 16)**	**Group K(n = 17)**	**Group S(n = 17)**	**ANOVA*P* value**	**Bonferroni-corrected post-hoc *P* values**		
					**D** **vs.** **K**	**D** **vs.** **S**	**K** **vs.** **S**
**1** **h**	6.81±0.75 (6.41-7.21)	7.59±0.62 (7.27-7.91)	7.94±0.56 (7.66-8.23)	**<0.001**	**0.003**	**<0.001**	0.350
**6** **h**	4.75±1.34 (4.04-5.46)	4.94±0.75 (4.56-5.33)	5.65±0.93 (5.17-6.13)	**0.038**	1.000	**0.048**	0.154
**12** **h**	3.50±1.26 (2.83-4.17)	3.71±0.77 (3.31-4.10)	4.82±1.13 (4.24-5.41)	**0.002**	1.000	**0.003**	**0.012**
**24** **h**	2.31±0.95 (1.81-2.82)	2.88±0.86 (2.44-3.32)	3.47±1.28 (2.81-4.13)	**0.010**	0.374	**0.008**	0.323

Values are expressed as mean ± standard deviation. Overall, intergroup comparisons were performed using one-way ANOVA, followed by Bonferroni-corrected post-hoc pairwise comparisons. A *P* value <0.05 was considered statistically significant.

D, dexmedetomidine; K, ketamine; S, saline; ANOVA, analysis of variance
